# Modelling chronotaxicity of cellular energy metabolism to facilitate the identification of altered metabolic states

**DOI:** 10.1038/srep29584

**Published:** 2016-08-03

**Authors:** Gemma Lancaster, Yevhen F. Suprunenko, Kirsten Jenkins, Aneta Stefanovska

**Affiliations:** 1 Department of Physics, Lancaster University, Lancaster, LA1 4YB, UK; 2 Institute of Integrative Biology, University of Liverpool, Liverpool, L69 7ZB, UK; 3 Randall Division of Cell & Molecular Biophysics, King’s College London, London, WC2R 2LS, UK

## Abstract

Altered cellular energy metabolism is a hallmark of many diseases, one notable example being cancer. Here, we focus on the identification of the transition from healthy to abnormal metabolic states. To do this, we study the dynamics of energy production in a cell. Due to the thermodynamic openness of a living cell, the inability to instantaneously match fluctuating supply and demand in energy metabolism results in nonautonomous time-varying oscillatory dynamics. However, such oscillatory dynamics is often neglected and treated as stochastic. Based on experimental evidence of metabolic oscillations, we show that changes in metabolic state can be described robustly by alterations in the chronotaxicity of the corresponding metabolic oscillations, i.e. the ability of an oscillator to resist external perturbations. We also present a method for the identification of chronotaxicity, applicable to general oscillatory signals and, importantly, apply this to real experimental data. Evidence of chronotaxicity was found in glycolytic oscillations in real yeast cells, verifying that chronotaxicity could be used to study transitions between metabolic states.

Cellular energy metabolism encompasses many processes, ultimately resulting in the production of adenosine triphosphate (ATP), the fuel continuously used by cells for many essential functions, such as maintenance of ionic balance across the plasma membrane, signalling, and protein synthesis. Every day, we turnover the equivalent of our body weight in ATP^1^, thus it is important to understand every stage of energy metabolism. Novel imaging techniques have provided insights into the function of metabolic pathways^2^,^3^,^4^,^5^, and have led to the growing understanding that many diseases can be associated with dysfunctional mechanisms of ATP production^6^,^7^,^8^,^9^,^10^. Increasing evidence suggests that metabolic dysfunction plays a key role in carcinogenesis^6^,^7^,^8^,^11^,^12^. Moreover, most other properties observed in cancer cells can be explained as consequences of this dysfunction^9^. Therefore, the observation of the cell in a state of metabolic transition may aid in the understanding of the effects of metabolism on the carcinogenic potential of the cell. Here, we study the dynamics of energy production, and investigate the possibility of identifying robust characteristics which can be (a) used to identify alterations in the metabolic state of a cell, (b) reliably identified from the observed metabolic dynamics.

The hypothesis that such a robust characteristic exists is based on a number of experimental observations of common patterns in metabolic dynamics which are distinctive in different metabolic states, suggesting that we may be able to identify a transition from a healthy or altered states by observing the properties of the dynamics of cellular metabolism.

First of all we focus on the oscillatory and time-dependent nature of the dynamics of energy metabolism. Indeed, the energy produced by a cell continuously fluctuates due to rate constants involved in the production and use of energy. Recently developed experimental techniques for the observation of energy metabolism^3^,^13^,^14^ clearly illustrate these fluctuations, mainly as oscillations, through the measurement of glycolytic intermediates, such as nicotinamide adenine dinucleotide (NADH), and the mitochondrial membrane potential (Δ*ψ*_*m*_). Although the oscillatory nature of energy metabolism is now more often appreciated^3^,^15^,^16^,^17^,^18^,^19^,^20^,^21^,^22^,^23^, metabolic oscillations can still be overlooked due to intercellular heterogeneity^24^ or considered as purely stochastic fluctuations^25^. Oscillations in glycolysis have long been observed in many types of cells, including yeast^26^, pancreatic *β* cells^27^ and muscle cells^16^. Observing NADH via fluorescence imaging^3^,^20^ provides an opportunity to observe the oscillatory dynamics of glycolysis. Mitochondrial oscillations have also been demonstrated. In yeast, in aerobic conditions, oscillations in Δ*ψ*_*m*_ were observed, and it was concluded that these were probably entraining the whole metabolic network of the cell^28^. As well as being oscillatory, energy production within a cell is inherently time-dependent. The contribution of each metabolic pathway to the cellular ATP supply depends on cell type and the current energy requirements of the cell, and is thus necessarily time-varying. This openness of the system of cellular energy metabolism inevitably leads to nonautonomous or time-dependent dynamics^29^,^30^.

Energy production via different metabolic processes is tightly regulated. Each metabolic state of the cell will be characterised by different pathways of ATP production. This will result in clear differences between healthy and altered states, arising from the cell switching between glycolytic and mitochondrial ATP production as a primary source of energy. A widely observed example of this is the metabolic switch to glycolysis for an increased proportion of energy production in cancer cells, even in the presence of oxygen. This is known as the Warburg effect^31^,^32^. These experimental observations, of metabolic oscillations and switches between metabolic states, suggest that we may be able to identify whether cells are in healthy or altered states by observing the properties of their oscillations.

Metabolic oscillations observed in glycolysis and in the mitochondria are coupled and can influence each other depending on the state of the cell^20^,^21^,^27^,^33^. This was demonstrated in the form of a driving influence from glycolysis on mitochondrial oscillations in semi-anaerobic conditions^20^ and at near anoxia^21^. This driving influence of glycolysis in these ‘altered’ states suggests that changes in this driving would accompany changes in the state of the cell. We propose that these characteristics of metabolic state can be considered under a newly introduced theoretical framework named chronotaxicity^34^. Chronotaxicity^35^,^36^ was recently introduced to describe physical properties of oscillatory systems which are inherently driven, and are capable of resisting perturbations. In doing so, they often generate complex time-dependent behaviour. Their ability to resist perturbation can be robustly identified regardless of the level of complexity, which makes chronotaxic systems an ideal model for cellular metabolic oscillations. We propose that observations of the driven metabolic oscillations imply that they are chronotaxic.

It was previously shown that chronotaxicity can be identified in a single time series^37^. This allowed us to test this hypothesis in real experimental data recorded by Gustavsson *et al*.^38^. Using these recordings of oscillatory glycolysis in single yeast (*S. cerevisiae*) cells, we investigated the chronotaxic properties of glycolytic oscillations. Yeast cells have been shown to have metabolic similarities with cancer cells^39^, and their metabolism has received much attention. Therefore we pay particular attention to the properties of yeast in the following work.

Here, we demonstrate that glycolytic oscillations in real yeast cells show clear signs of chronotaxicity. Based on this, we propose chronotaxicity as a robust characteristic whose alterations signify changes in energy metabolism, facilitating identification of altered states in the cell. A theoretical model of different metabolic states is developed and the interplay between glycolytic and mitochondrial involvement in ATP production within a cell is investigated. We also consider how metabolic oscillations may be driven by factors such as substrate availability.

The model shows how the temporal evolution of different states of cellular energy metabolism could be described based on changes in the robustness to external perturbations, or chronotaxicity. This provides a potential method of observing metabolic switches in real cells and investigating possible links to carcinogenesis.

## Results

### Identifying chronotaxicity in real metabolic oscillations

Glycolysis in yeast cells is one of the most widely studied and well characterised biological oscillators. It was previously thought that glycolytic oscillations only arose as a result of synchronization between yeast cells, but recently Gustavsson *et al*.^38^,^40^ demonstrated glycolytic sustained oscillations in individual isolated yeast cells. Here, to investigate the applicability of chronotaxicity to metabolic oscillations we use data recorded by Gustavsson *et al*.^38^ and test it for chronotaxicity using phase fluctuation analysis (PFA), as described in Methods.

**Figure 1:**
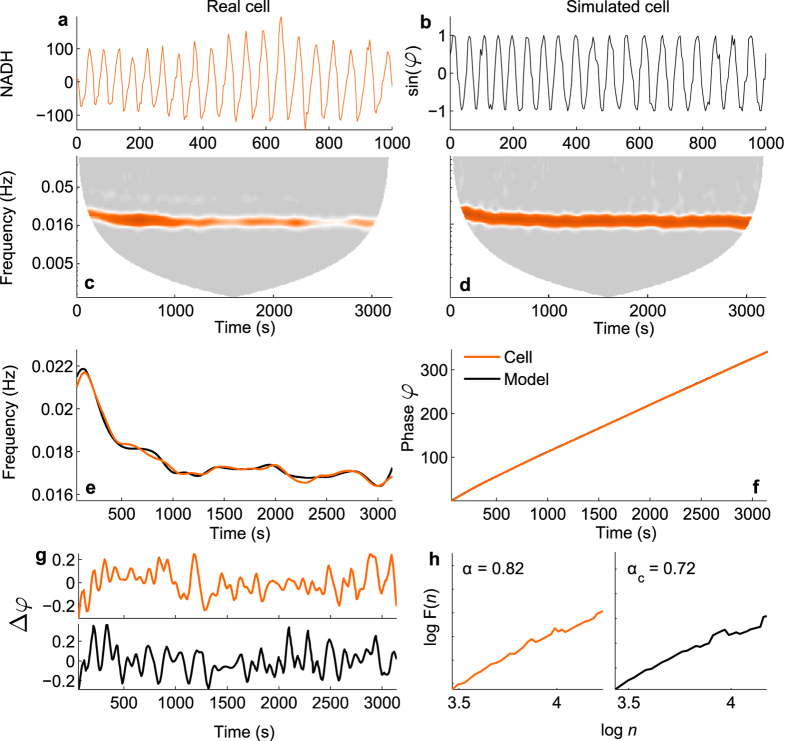
Testing real experimental data for chronotaxicity. (**a**) Example NADH signal from an isolated yeast cell, recorded in^38^. (**b**) Time series of sin*φ*_*GO*_ from the model using the instantaneous frequency extracted from (**a**) as the driver *ω*_*G*_. (**c**,**d**) Continuous wavelet transforms of the time series in (**a**,**b**), respectively. (**e**) The instantaneous frequency of the oscillatory modes were extracted from the wavelet transforms, and smoothed using a moving average. (**f**) Integrating over the smoothed frequency provides the phase for each case. (**g**) Subtracting the smoothed phase from the observed phase provides the phase fluctuations, Δ*φ*, in the system. (**h**) Detrended fluctuation analysis performed on Δ*φ* suggests that the glycolytic oscillations observed in a real yeast cell (orange line) are chronotaxic. *α* = DFA exponent from yeast cell, *α*_*c*_ = DFA exponent from model.

NADH data obtained by Gustavsson and co-authors^38^ was available from 34 yeast cells, 6 of which did not demonstrate visible oscillations so were excluded from the analysis. An example NADH signal can be seen in Fig. 1a. It was previously demonstrated that at least 30 cycles of oscillation are required to test for chronotaxicity^41^, therefore all cells which did not meet this requirement were excluded. 16 cells met this criteria. Phase fluctuation analysis (PFA)^37^ was then applied to the remaining NADH signals to determine whether the oscillations observed in these cells exhibited hallmarks of chronotaxicity. The mean value of *α* for the 16 included cells was 0.768, which characterises oscillations as chronotaxic. An example analysis on a real yeast cell can be seen in Fig. 1. This demonstrates that real metabolic oscillators are able to be chronotaxic in certain states. Particularly, as shown here, this is the case when metabolic oscillations are sustained and stable despite noise and perturbations. The identification of these biological oscillations as chronotaxic demonstrates that the concept of chronotaxicity, originating from the theory of nonautonomous dynamical systems^29^,^30^, can be used to describe real biological oscillations in cellular energy metabolism. Therefore, inverse approach methods may be utilised to detect chronotaxicity with no prior knowledge of the system, as is the case in real experimental data. This result allows us to apply the concept of chronotaxicity to cellular energy metabolism in general.

### Modelling metabolic oscillations and their chronotaxicity

Based on experimental observations of sustained oscillations, and the evidence of chronotaxicity highlighted in yeast cells, we now consider the chronotaxicity of cellular energy metabolism in healthy and ‘altered’ states, and how this information could be used to observe transitions between these states over time. We consider chronotaxicity during the metabolic transition of a single cell from a state in which aerobic respiration provides the majority of ATP, to one where the cell is increasingly reliant on glycolysis. We consider the former as a healthy state, and the latter as an undesirable state for most cells. An upregulation of glycolysis may be observed temporarily in intermittent hypoxia, or consistently in cancer or yeast cells. Whilst a sign of stress in hypoxic cells, normal yeast cells actually demonstrate a preference for a glycolytic metabolism, displaying many similar metabolic features to cancer cells^39^,^42^.

**Figure 2:**
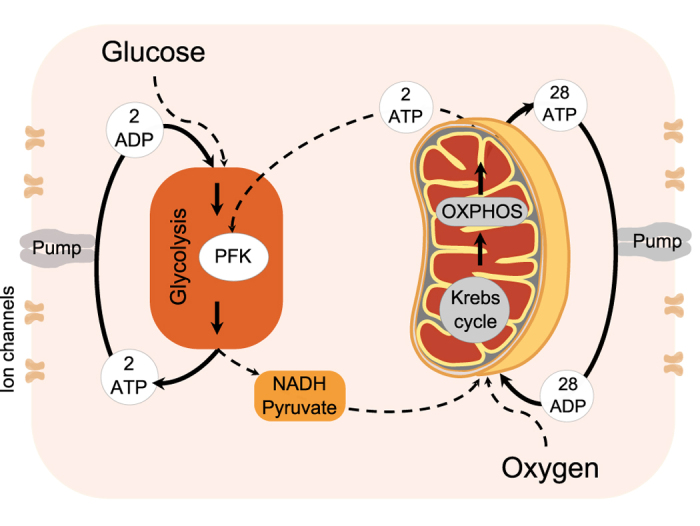
Cellular energy metabolism. ATP production in a cell (thick lines) occurs via glycolysis using glucose, and when oxygen is present ATP is produced via oxidative phosphorylation (OXPHOS) using the products of glycolysis. Both processes are oscillatory, and can influence each other (for details see main text). Couplings between these processes as well as external influences are shown by dashed lines.

Firstly, we discuss the important features of cellular energy metabolism relevant to the model (see Fig. 2). ATP production begins with glycolysis, which converts glucose to pyruvate and produces ATP and nicotinamide adenine dinucleotide (NADH). Pyruvate is then used during pyruvate decarboxylation, the products of which are used in the Krebs cycle. This releases more ATP and leads to production of substrates (including NADH) which are used to create a hydrogen ion gradient across the inner mitochondrial membrane. This gradient then drives the production of ATP from ADP and inorganic phosphate, in the process of oxidative phosphorylation (OXPHOS). The activity of each of these pathways, and the extent to which the cell relies on them for ATP production depends upon many factors, including the current energy requirements of the cell, and the speed with which each process can match demand. For example, aerobic respiration is much more efficient in terms of amount of ATP produced than glycolysis alone, but glycolysis produces ATP at a faster rate.

In the model which follows, we identify glycolytic and mitochondrial oscillators, and simplify the metabolic pathways and interactions between oscillators to only include features which are necessary to capture chronotaxicity, which can describe subtle changes in dynamics due to the change in the interactions between metabolic oscillators.

#### Glycolytic oscillator

Oscillations in glycolysis have long been observed in many types of cells, including yeast^26^, pancreatic *β* cells^27^ and muscle cells^16^. Glycolytic oscillations have been shown to provide an advantage in maintaining a high ATP/ADP ratio^43^,^44^. The source of these oscillations is still debated, but evidence suggests that a reaction involving phosphofructokinase (PFK) is responsible for their origin^3^,^33^. In addition to PFK, another time-dependent factor which influences the dynamics of glycolysis is the availability of glucose. Reijenga *et al*. demonstrated that the frequency of glycolytic oscillations is directly affected by sugar transport^45^, with a maltose induced decrease in sugar transport resulting in a decrease in the frequency of glycolytic oscillations.

The effects of the inevitable fluctuations in the availability of glucose *in vivo* was further explored^46^ through simulation of fluctuating external glucose, and it was found that the glycolytic dynamics of yeast cells that do not usually exhibit intrinsic oscillations oscillated at the frequency of extracellular glucose pulsing, demonstrating a clear driving influence. Boiteux *et al*. investigated the effects on oscillatory glycolysis of stochastic, periodic, and steady sources of substrate^47^, and found that stochastic variation of the rate of glucose input leads to sustained periodic behaviour, with small variations in period around a preferred frequency, i.e. it exhibits stability but with a time-varying frequency. Periodic variations of glucose in this study revealed instantaneous frequency responses and harmonic entrainment at specific frequencies. More recently, the ability to measure metabolic dynamics in individual yeast cells has allowed the investigation of the effects of periodic external perturbations on existing glycolytic oscillations^40^. This study found that oscillatory cells synchronize through externally induced phase shifts alone, whilst non-oscillating cells also require amplitude changes to exhibit externally driven oscillations.

Based on the observed oscillations in glycolysis, and the inevitable time-variability of glucose availability *in vivo*, we consider glycolysis as a single oscillator, the glycolytic oscillator (GO), which may be influenced by external glucose levels.

#### Mitochondrial oscillator

Mitochondrial oscillations have been associated with cycles of oxidation and reduction of the intracellular NADH pool and extramitochondrial factors. In cases of oxidative stress or substrate deprivation in cardiac myocytes, the inner mitochondrial membrane potential may destabilise, causing depolarization and oscillation^14^,^22^, forcing synchronous oscillations within the mitochondrial network^13^,^14^,^17^. Mitochondrial oscillations are usually observed in the form of Δ*ψ*_*m*_ oscillations^19^ using fluorescence^20^.

As is effectively assumed when observing Δ*ψ*_*m*_ as a measure of mitochondrial metabolic activity, we combine the Krebs cycle and OXPHOS, both of which occur in the mitochondria, into a single metabolic oscillator, the mitochondrial oscillator (MO), which may be driven internally or by the extramitochondrial factors such as the plasma membrane potential or Ca2+^19^.

#### Interactions between metabolic oscillators

Interactions between mitochondrial and glycolytic oscillations have been experimentally demonstrated under metabolic stress in cardiac myocytes^21^, and in yeast cells^20^. Both studies concluded that under conditions in which the cell is relying on glycolysis for the majority of ATP production, which can be considered as an altered state, oscillations in glycolysis drive those in Δ*ψ*_*m*_. They also demonstrated the indirect influence of mitochondrial ATP production on glycolysis even in this state, via sensing of the ATP/ADP ratio, i.e. the lower this ratio, the harder glycolysis has to work. This link is provided by the reversal of the inhibition of PFK in the glycolytic oscillator^33^, schematically shown Fig. 2. In the healthy state, the majority of ATP will be produced via OXPHOS, and glycolysis will be suppressed to the level necessary to provide substrates for further metabolic reactions. Glycolysis will still be able to influence mitochondrial processes through the availability of pyruvate and NADH, but metabolic oscillations will likely be driven by the mitochondrial oscillations^28^.

It is the *interactions* between these oscillators (MO and GO) which would change in a transition to an altered metabolic state. Therefore, our model provides sufficient detail to capture this transition, by considering only these interactions (see Fig. 2). All other possible interactions, and specific enzymatic pathways, are not considered.

#### Chronotaxicity of metabolic oscillators

In measurements of oscillations in healthy and altered metabolism an apparent stability of glycolytic and mitochondrial oscillations has been observed^3^,^13^,^33^. These oscillations appear to be time-variable yet very stable^14^,^22^ despite noise and perturbations, suggesting the presence of chronotaxicity in the system.

**Figure 3:**
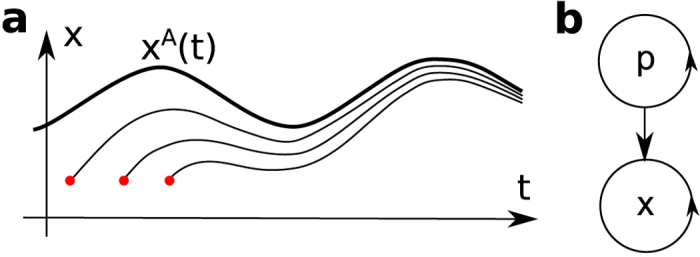
Chronotaxic system. (**a**) Time-dependent point attractor **x**^*A*^(*t*), whose trajectory is shown as a thick line, is the main characteristic of a chronotaxic system. Trajectories with different initial conditions (red dots) are approaching **x**^*A*^(*t*). (**b**) The simplest configuration of dynamical system where a response system **x** may be chronotaxic due to a drive system **p**. Detailed definition of chronotaxic systems is given in Methods.

The chronotaxicity of GO and MO may be realized in two general configurations. First, the required drive system (see the definition of chronotaxic systems in Methods) could be provided by any *external* oscillatory influence to GO and MO strong enough to create chronotaxicity. Particularly, oscillators may be chronotaxic because of interaction with other metabolic oscillators^20^, or due to interaction with other external oscillations such as fluctuating external glucose^27^,^46^ or oxygen. In the GO, we consider the time-varying availability of glucose as the potential cause of chronotaxicity, i.e. as the drive system in Fig. 3b. Similarly, we may consider dynamical oxygen availability as the drive system of the MO. However, if glucose and oxygen are abundant within the cell, they are unlikely to be the driver for each process.

In the second configuration, the chronotaxicity could be created due to the *inner* structure of the oscillator, i.e. we assume that due to finite reaction times and interactions within structural elements of the oscillator the stable time-dependent point attractor could appear. Importantly, the oscillations observed by Gustavsson *et al*.^38^ in isolated yeast cells, whilst occurring with similar frequencies, were not synchronized. As the external glucose level in these experiments was constant, the oscillations must occur as a result of an internal mechanism, which is presented as a second configuration where chronotaxicity of metabolic oscillators may exist.

Hereafter we do not distinguish between these two configurations, and consider the external resources glucose and oxygen as the drivers which directly or indirectly define chronotaxicity of the GO and MO, independent of the exact mechanism by which this occurs. Our focus will be on the *changes* in chronotaxicity, i.e. alterations in the stability of metabolic oscillations in different states.

#### The model

Based on the above discussion, we consider cellular energy metabolism as a system of coupled oscillators, comprising the GO, the MO, and their respective external influences, which we name glucose (G) and oxygen (O), respectively. The inputs to the GO are considered to be glucose and mitochondrial ATP (ATP_MO_). The end products of glycolysis which are relevant to the model are glycolytic ATP (ATP_GO_) and NADH. The MO is regulated by the availability of substrates. Here, we consider the availability of NADH as the primary influence on the mitochondrial oscillator (see Fig. 2). The final output of both oscillators represents oscillatory ATP. ATP concentration was previously shown to be oscillatory in yeast cells by Özalp *et al*.^48^.

Chronotaxicity is defined by the presence of a time-dependent steady state, and thus does not depend on the particular shape of the oscillations in a signal, only on how their frequency varies in time. Therefore, it can be robustly identified from phase dynamics alone, even when the amplitude dynamics is complex^37^. We therefore model this system using coupled phase oscillators,


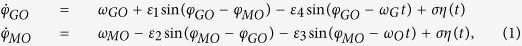


where *φ*_*GO*_ and *φ*_*MO*_ are the instantaneous phases of the GO and MO, respectively, *ω*_*GO*_ and *ω*_*MO*_ are the natural frequencies of the GO and MO, *ω*_*G*_ and *ω*_*O*_ are the frequencies of the external drivers and *η*(*t*) is white Gaussian noise. Considering that the GO and MO are continuously interacting, as discussed above, they are represented as bidirectionally coupled oscillators, with coupling strengths *ε*_1_ and *ε*_2_ which may vary to represent different metabolic states. The inhibitory nature of the influence of ATP_MO_ production on ATP_GO_ production is represented by a repulsive coupling *ε*_1_, whilst the excitatory nature of glycolytic oscillations on mitochondrial oscillations is represented by an attractive coupling *ε*_2_. The external influences on the GO and MO are represented as unidirectionally coupled drivers, with coupling strengths *ε*_3_ and *ε*_4_ for O and G respectively, which may also vary.

**Figure 4:**
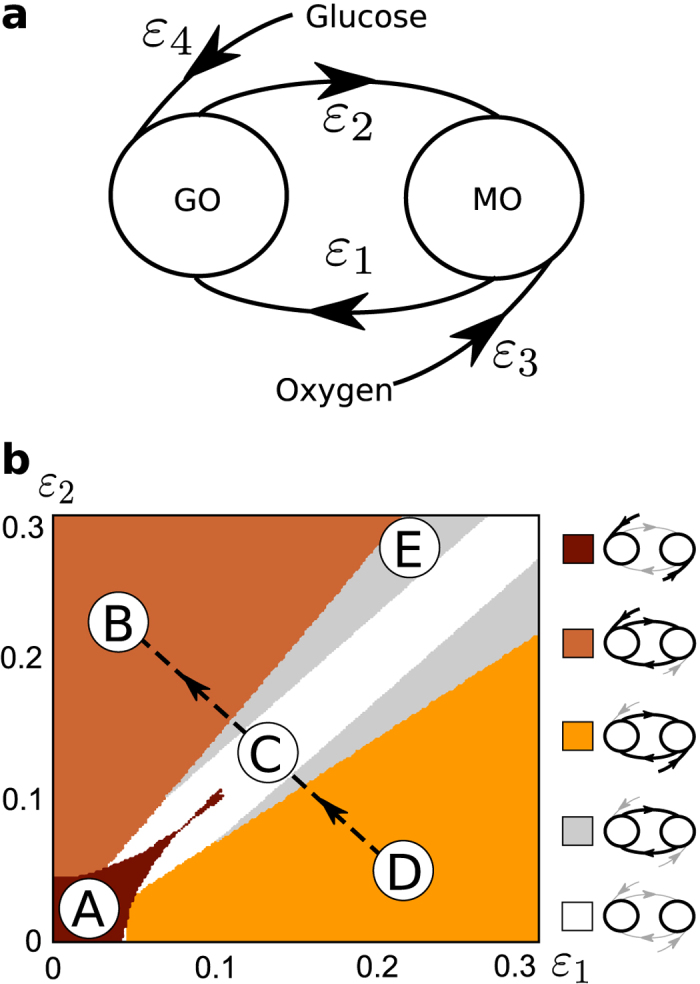
Model (1) of metabolic oscillations in a cell, and different types of dynamics and the transition from healthy to an altered metabolic state. (**a**) The glycolytic and mitochondrial oscillators (GO and MO, respectively) are represented by two phase oscillators coupled to each other via repulsive *ε*_1_ (due to the inhibitory nature of influence of MO on GO) and attractive coupling *ε*_2_ (due to excitatory influence of GO on MO). Chronotaxicity arises through attractive couplings *ε*_3_ and *ε*_4_ to glucose and oxygen drivers. (**b**) In numerical simulations, couplings *ε*_1_ and *ε*_2_ were varied to represent different metabolic states. Chronotaxicity of the system was tested for each pair of couplings by checking synchronization between each metabolic oscillator and its potential drivers, see Supplementary Methods. Various types of dynamics of equations (1) with different chronotaxicities were revealed. Region A (dark brown): GO and MO are synchronized with their drivers (Glucose and Oxygen), but not with each other (both GO and MO are chronotaxic); Region B (orange): GO and MO are both chronotaxic and synchronized with the Glucose driver; Region C (white): GO and MO are both not chronotaxic and not synchronized with anything; Region D (yellow): GO and MO are both chronotaxic and synchronized with the Oxygen driver; Region E (light gray) GO and MO are both nonchronotaxic and synchronized only with each other. Regions F and G (not shown, see Supplementary Figs S1 and S3): only GO or only MO is chronotaxic respectively. A potential transition of the system from healthy to a potentially carcinogenic state could correspond to dynamical changes from region D to region B, which are both chronotaxic, but with different drivers, via regions C and E, which are non-chronotaxic. For simplicity, *ε*_3_ and *ε*_4_ were considered to be equal (*ε*_3_ = *ε*_4_ = 0.025), but can easily be changed depending on the exact metabolic state one needs to model, e.g. specific substrate deprivations (see Supplementary Fig. S2). Supplementary Figs S3 and S4 show the effects on the system of different constant frequencies and time varying frequencies, respectively.

The chronotaxic dynamics of this model was tested numerically. The system (1) was integrated with varying parameters. Numerically, for an oscillator to be chronotaxic, it was required that the observed oscillator was synchronized to one of the unidirectionally coupled drivers G or O. Using this test, 7 different types of dynamics were revealed, the most relevant 5 regions shown in Fig. 4b. Example phase trajectories for all types are shown in Supplementary Fig. S1. Approximate frequencies of these oscillations in different regions are summarised in Table 1.

**Figure 5:**
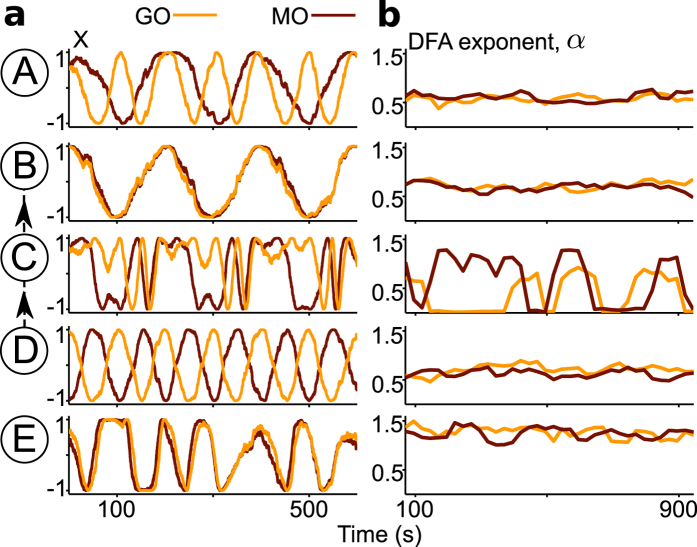
Identification of chronotaxicity via inverse approach methods. (**a**) Examples of dynamics from regions A–E, as defined in Fig. 4b. The additive white Gaussian noise with σ = 0.05 was used. (**b**) Detrended fluctuation analysis (DFA) exponents *α* characterising chronotaxicity in each region: for *α* ∈ (0.5, 1) the system can be considered as chronotaxic, for *α* ∈ (1, 1.5) the system can be considered as non-chronotaxic. In region C the exponent *α* changes too fast, and the DFA method is not applicable, however such dynamics of *α* suggest that system is not chronotaxic. This inverse approach shows very good agreement with the chronotaxicity tests directly from the model, revealing that metabolic dynamics in regions A, B and D are chronotaxic, while regions C and E are not. Thus, it may be used to identify chronotaxicity in real data, using a single time series. To demonstrate the method, the time series used here contain at least 100 cycles of oscillation. In reality, this number of cycles is not always feasible. However, this method may still be applied on shorter time series, with reliable results, see Supplementary Fig. S5.

In real data, utilising chronotaxicity as the defining parameter of the system is superior to the consideration of synchronization alone, as it can be identified experimentally from any single time series, whereas synchronization requires measurements of all interacting oscillators. Therefore, in this case, it would be sufficient to have measurements of only ATP_GO_ or ATP_MO_ to determine their chronotaxicity. To demonstrate this, phase fluctuation analysis was applied to the generated dynamics of GO and MO using parameters from each region shown in Fig. 4b. The chronotaxicity of each metabolic oscillator was tested separately. PFA was used to characterise the phase fluctuations for each oscillator in each region (see Fig. 5). Excellent agreement is shown between the chronotaxicity as calculated by the model using synchronization conditions, and chronotaxicity as calculated via the inverse approach with no prior knowledge. This illustrates that the method is applicable to the observation and identification of chronotaxicity in real systems, where the dynamics are unknown beforehand.

### Applicability of the model: modelling chronotaxicity observed in experimental yeast data

**Figure 6:**
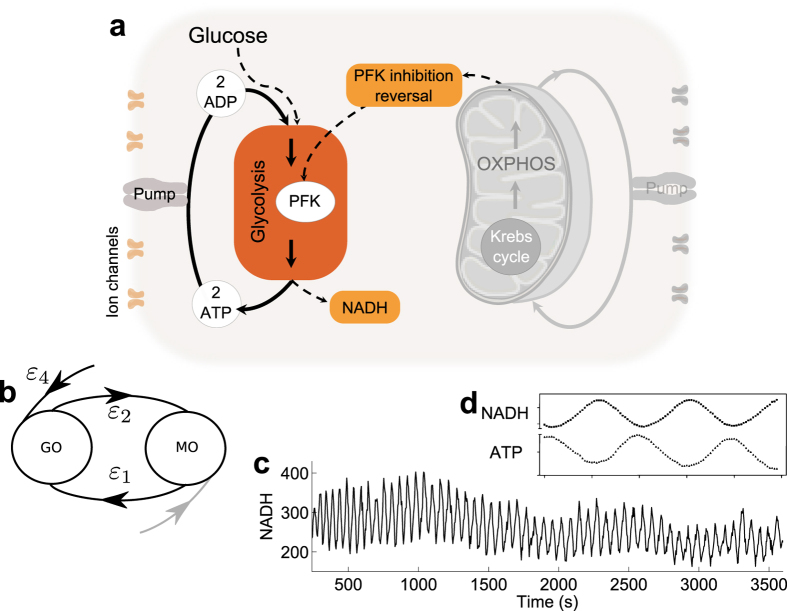
Verifying the glycolytic oscillator (GO) with real experimental data. (**a**) The yeast data recorded from individual yeast cells by Gustavsson *et al*.^38^ provides our ‘altered’ metabolic state. The addition of glucose followed by cyanide to glucose starved yeast cells induces glycolytic oscillations by preventing respiration. Therefore, glycolysis is upregulated by the reversal of PFK inhibition in response to the reduction in ATP_MO_. (**b**) The situation described in (**a**) can be represented by our model as the dynamics of the system being driven by the glucose driver, causing the system to be chronotaxic. (**c**) Example NADH time series from an isolated yeast cell exhibiting glycolytic oscillations. (**d**) Comparison of NADH and ATP oscillations in yeast glycolysis shows that they oscillate 180 out of phase^48^,^49^. Modified from^49^.

The applicability of the model is tested on the data of NADH recorded by Gustavsson *et al*.^38^. For this we utilise the closely linked dynamics of intracellular ATP and NADH arising from glycolysis. It has been shown previously that both ATP and NADH are oscillatory in yeast cells during glycolytic activity, and that these oscillations have the same frequency but are out of phase by around 180° 48,49, as demonstrated in Fig. 6d. This means that measurements of NADH in yeast cells may be used to provide an approximation of ATP dynamics. Although the amplitude of these parameters will differ, their phase relationship will remain the same, and can thus be represented by our phase oscillator model. Using this information, the model can be tested for the case of the glycolytic oscillator based on NADH measurements, which are more readily available.

In the experiments by Gustavsson *et al*.^38^. NADH in individual yeast cells was shown to oscillate following starvation and the addition of cyanide. In this state, glycolysis can be the only means of energy production, as cyanide halts respiration, effectively removing the effects of the mitochondrial oscillator (MO). It is therefore expected that the metabolic state induced in this experimental setup should correspond to our ‘altered’ state, i.e. region B in Fig. 4b, where glycolytic oscillations drive the dynamics of the system, and the GO is chronotaxic.

The model was used to numerically simulate the GO in a chronotaxic (*ε*_1_ = 0, *ε*_2_ = 0, *ε*_3_ = 0, *ε*_4_ = 0.25, σ = 0.2) state using the instantaneous frequency extracted from the real experimental data. To make the system chronotaxic, the extracted frequency was used as the driver *ω*_*G*_ of the glycolytic oscillator. This provided a chronotaxic oscillator with the same oscillation frequency as the experimental data, allowing the DFA exponent *α* to be compared between cases (see Fig. 1). Due to the relatively short recording time causing variation between simulations, they were repeated 3 times and the average value of *α* taken. The mean value of *α* for the chronotaxic simulations was 0.772 , compared to 0.768 which is the mean value of *α* for the 16 included cells recorded by Gustavsson *et al*.^38^.

The distributions of exponents did not significantly differ between those calculated in the cells and those calculated in the simulated chronotaxic system (*p* = 0.86) as calculated using the Wilcoxon ranksum test. This shows that our model, although simple, incorporates enough features to allow the calculation of the presented characteristic, chronotaxicity, and that evidence of this characteristic appears to be present in yeast glycolytic dynamics. This verifies the applicability of our model to the glycolytic oscillator. Further investigation is required into the mitochondrial oscillator, and other metabolic states.

### Transition between healthy and altered metabolic states in the model

To identify metabolic states, we consider an altered state, such as the glycolysis dependent and potentially carcinogenic case discussed above, to correspond to the dynamics of the model in which the phase of a chronotaxic GO entrains the phase of MO, i.e. region B in Fig. 4b. In contrast, the normal state may correspond to the case where the phase of MO entrains the phase of GO, as discussed in ref. 28. Therefore, the normal state corresponds to region D (where oscillators are chronotaxic with oxygen as a driving system). However, it may also be possible that in the normal state both oscillators are chronotaxic due to only their external influences (from oxygen and glucose), with the interactions between MO and GO not strong enough for entrainment, as shown in region A. In all chronotaxic cases, phase fluctuation analysis will return a DFA exponent around 0.5 < *α* < 1. The evolution of the system from the normal to the altered state is from D to B via regions C and E, regions which are both non-chronotaxic, and will be characterised by a DFA exponent around 1 < *α* < 1.5, allowing the observation of the transition between states.

## Discussion

This work is based on experimental evidence of metabolic oscillators^3^,^13^,^15^,^19^,^22^,^23^ and their interactions^20^,^21^,^27^,^33^. In experimental studies oscillations are often overlooked^24^, as several oscillations may contribute to the same signal thus almost cancelling each other despite existing separately. Alternatively, due to their highly complex nature they are often treated as stochastic^25^. Even in cases when oscillations and the interactions between them have been studied, the exact characteristics of their amplitude and phase relations have not been considered. Several studies of mitochondrial and glycolytic oscillations have shown that fluctuations of ATP production in a cell are not fully stochastic, but have nonautonomous and deterministic oscillatory components^3^,^13^,^14^,^15^,^16^,^17^,^18^,^19^,^20^,^21^,^22^,^23^,^26^,^27^,^33^,^45^. Therefore, these oscillations reflect underlying deterministic processes.

Here we have proposed a measurable characteristic of metabolic oscillations, chronotaxicity, which is expected to change during the transition of a healthy cell to a state with altered energy metabolism. This was demonstrated using a qualitative phase oscillator model of metabolic oscillations. The model captures only the most general and universal oscillatory dynamics and interactions, and thus provides the advantage that it is applicable to oscillatory metabolism in general, independent of cell type. The model explicitly takes into account the openness of cellular energy metabolism, and therefore its nonautonomous dynamics. The model, based on the theory of chronotaxic systems, introduces a new approach in which the complex metabolic system is considered as a set of functional rather than structural units, these functional units being interacting glycolytic and mitochondrial oscillators with chronotaxic properties.

As a concept within the theory of dynamical systems, describing oscillators which are driven by other oscillators, it is not trivial that chronotaxicity can be applied to biological oscillators, and that the driving in biological oscillators represents itself in the same way as in dynamical systems. In this work, we investigated the applicability of the concept of chronotaxicity to real biological oscillations observed in real experimental studies of cellular metabolism. We proved our assumptions that biological oscillators can be chronotaxic, and that their chronotaxicity can be identified from real experimental observations, and developed a model of cellular energy metabolism.

The interactions of oscillatory processes can be between amplitude and amplitude, phase and phase, or phase and amplitude. These possibilities show how many scenarios of regulations of physiological functions may occur. In this paper, for the sake of simplicity, we have selected phase oscillators and restricted the discussion to phase-phase interactions. Nevertheless, in observations of real life systems the chronotaxicity can be identified from phase dynamics alone, even when amplitude dynamics is complex^37^, thus making our model verifiable by experimental observations. Moreover, by identifying the characteristics of chronotaxicity we have shown that one in principle can detect a transition to a carcinogenic state of cellular functioning, provided metabolic oscillations are present in both states of the cell. The state in which the glycolytic oscillator determines the dynamics of energy metabolism, or the ‘altered’ state, was experimentally verified using real data from yeast cells exhibiting glycolytic oscillations. The described inverse approach methods found evidence of chronotaxicity in this case, as expected from the numerical simulations of the model.

Cellular metabolism may be affected by many more processes and interactions than those considered here. The simplicity of the model could easily facilitate the inclusion of further couplings, for example the consideration of calcium dynamics or genetic factors in energy production. Calcium has been shown to directly influence mitochondrial dynamics via many pathways^50^,^51^, while genetic mutations can have a direct effect on mitochondrial function^52^. These effects could be included in the model as influences to couplings, extra oscillators, or adaptations of the external drivers.

Results presented in this paper set up bases for experimental verification of the hypothesis that chronotaxicity can be used to identify transitions between metabolic states in a cell, for example the metabolic switch observed in cancer cells. We have demonstrated evidence of chronotaxicity in real metabolic oscillations, which led to a new way of studying metabolic processes inside a cell. The model provides a framework within which the existing understanding of biochemical reactions involved in metabolic processes along with new observations based on recently introduced functional imaging methods^2^,^3^,^4^,^5^ can be unified in a single picture. Furthermore, focusing on the transitions between metabolic states could facilitate the development of new therapeutic strategies.

## Methods

### Glycolytic oscillations in isolated yeast cells

As a real life example of driven metabolic oscillations we use glycolytic oscillations in individual isolated yeast cells recorded by Gustavsson *et al*.^38^. The brief description of the experiments setup is presented below, for more details see the original work by Gustavsson and co-authors^38^. *Saccharomyces cerevisiae* cells were harvested at the time of diauxic shift, starved of glucose for 3 hours and then stored at 0–4 °C until use. The cells were then placed in a microfluidic chamber at a distance of ~10 *μ*m apart using optical tweezers. The cells were covered with 20 mM glucose solution for 4 minutes before flows were increased in order to cover the cells with 20 mM glucose/5 mM KCN solution. NADH fluorescence was monitored for 60 minutes at a sampling frequency of 0.25 Hz.

### Characterising driven oscillators with chronotaxicity

The class of chronotaxic systems identifies oscillatory dynamical systems with dynamics ordered in time (*chronos* – time, *taxis* – order)^34^. Such ordering is typical for driven oscillators, where the drive system determines the dynamics of a response system. Chronotaxic systems can sustain their dynamics even with continuous external perturbations. Introduced for low-dimensional and high-dimensional dynamical systems^34^,^35^,^36^, chronotaxic systems have been shown to be useful in studies of living systems, one example being the application to the cardiorespiratory system^37^.

In addition to living and open systems, which are in continuous contact with the external environment, chronotaxic systems are nonautonomous dynamical systems^29^, i.e. their dynamics explicitly depends on time, as shown in the equation,


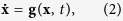


where **x** = (*x*_1,_ *x*_2_, …, *x*_*m*_) is a vector of coordinates which fully determines the state of a system at a given moment of time in phase space **x** ∈ R^*m*^. Alternatively, chronotaxic systems can be described by drive and response systems, as follows


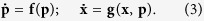


The main defining feature of chronotaxic systems is a time-dependent steady state of point attractor **x**^*A*^(*t*) (see Fig. 3a) which exists in the phase space of a chronotaxic system due to the influence from a drive system **p** (Fig. 3b). The trajectory **x**^*A*^(*t*) can be viewed as a uniformly hyperbolic trajectory^53^ which is linearly attracting in such a way that the distance between a neighboring trajectory and **x**^*A*^(*t*) can only contract in an unperturbed chronotaxic system. For more details and for relations between chronotaxic and other dynamical systems see ref. [36].

A simple example is given by unidirectionally coupled phase oscillators with phase *φ*_X_ driven by a phase *φ*_P_ as shown in the equation,


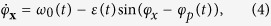


where 
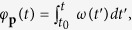
 and *ω*_0_ is the natural frequency of the observed oscillator, 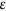 is coupling strength, and *ω* is the frequency of the driving oscillator. The time-dependent point attractor will exist if the condition of chronotaxicity^35^ is fulfilled, i.e. if 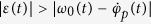 and if the coupling strength *ε*(*t*) does not change its sign. In the case of a particular choice 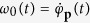 and *ε*(*t*) > 0, the time-dependent steady state 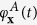 can be found analytically, 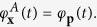 Chronotaxic systems in the simplest case could be considered as a generalization of synchronized unidirectionally coupled phase oscillators (phase-locked loops^53^).

Taking into account that the dynamical system **x** is chronotaxic due to the influence from the driver **p**, chronotaxicity will change when the external influence from **p** changes. This makes chronotaxicity a perfect candidate for investigation in the study of metabolic oscillators under changing driving influences.

### Detecting chronotaxicity from a single time series

One of main advantages of chronotaxicity is that it can be identified experimentally from a single time series, whereas otherwise the identification of drive-response relationship would require measurements of the driver as well as the response system. To identify chronotaxicity in a time series, a method named phase fluctuation analysis (PFA) was recently developed^37^. PFA involves the separation of the amplitude and phase of an oscillatory mode by extracting an estimated phase of the point attractor, *φ*^A*^, from the continuous wavelet transform^54^,


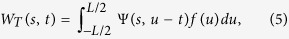


where Ψ(*s*,*t*) is the mother wavelet which is time-shifted according to *t* and scaled according to the parameter *s*. The oscillation can then be traced in *W*_*T*_(*s*, *t*). The instantaneous frequency of the oscillation at each time point can be estimated using either the synchrosqueezed wavelet transform^55^ or a ridge-extraction method^56^. The phase is then calculated by integrating over the instantaneous frequency in time. The estimation of angular velocity 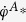 can be found by smoothing over the frequency extracted from the wavelet transform. Then, 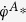 is integrated over to give *φ*^A*^.

In this approach, perturbations are assumed to be due to an uncorrelated Gaussian process. In chronotaxic systems, perturbations decay due to the influence of the point attractor and the divergence from the attractor is similar to the original Gaussian process^37^. In contrast, in non-chronotaxic systems, where the phase of oscillation is neutrally stable, the perturbations are integrated over, resulting in a random walk (i.e. Brownian noise). To distinguish these two cases, detrended fluctuation analysis (DFA)^57^ is performed on the phase fluctuations extracted from the time series. First, the difference Δ*φ* = φ^*^ − *φ*^A*^ is calculated to obtain information about the divergence of the system from the point attractor due to perturbations. The DFA technique explores the fractal self-similarity of fluctuations at different timescales in Δ*φ*. The scaling of fluctuations is determined by the self-similarity parameter *α*.

To estimate *α* the time series is integrated in time and divided into sections of length *n*. The local trend is removed for each section by subtracting a fitted polynomial, usually a first order fit^57^,^58^. The root mean square fluctuation *F*(*n*) for the scale equal to *n* is then defined by the following equation,


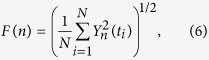


where *Y*_*n*_(*t*) is the integrated and detrended time series of length *N*. The self-similarity parameter is given by the gradient of the line of the plot of log*F*(*n*) against log*n*. The self-similarity parameter *α* for Δ*φ* for uncorrelated Gaussian noise (as expected in chronotaxic systems) gives a value of 0.5; in contrast, for integrated white Gaussian noise (as expected in non-chronotaxic systems) the parameter *α* returns a value of 1.5. In Fig. 5 the lowest length *n* was chosen to be equal to 2 cycles of oscillations, and the largest length *n* was chosen to be equal to 15 cycles of oscillations. Supplementary Fig. S5 shows how this method may be applied for an example ATP signal obtained from the model.

In order to reliably test for chronotaxicity, it should be noted that the time series should be sufficiently long, i.e. contain at least 30 cycles of oscillation (may vary depending on the characteristics of the data), be evenly sampled, and have a sampling frequency which is high enough to capture the dynamics at the frequency of interest^41^.

## Additional Information

**How to cite this article**: Lancaster, G. *et al*. Modelling chronotaxicity of cellular energy metabolism to facilitate the identification of altered metabolic states. *Sci. Rep*. **6**, 29584; doi: 10.1038/srep29584 (2016).

## Supplementary Material

Supplementary Information

## Figures and Tables

**Table 1:** Approximate characteristic frequencies of GO and MO in the regions shown in Fig. 4b.

	Glycolytic	Mitochondrial
A	*ω*_*G*_	*ω*_*O*_
B	*ω*_*G*_	*ω*_*G*_
C	*ω*_*GO*_	*ω*_*MO*_
D	*ω*_*O*_	*ω*_*O*_
E	ω^*^	ω^*^

In regions A, B, and D each oscillator is synchronized either to its own driver or to the other oscillator and its driver. In these cases, frequencies are influenced by driving oscillators. In C the oscillators are not synchronized to anything, hence the characteristic frequencies are determined by natural frequencies. In E oscillators are synchronized to each other only, hence the frequency ω^*^ corresponds to the frequency of a synchronized state assuming *ε*_3_ = *ε*_4_ = 0, and ω^*^ can be found from the condition of synchronization 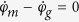, 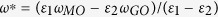.
