# Supplementary material for: Modelling chronotaxicity of cellular energy metabolism to facilitate the identification of altered metabolic states
Source: Sci Rep. 2016 Aug 3;6:29584. doi: 10.1038/srep29584 (PMC4971499; doi:10.1038/srep29584)
Supplement: Supplementary Information [file srep29584-s1.pdf]

Modelling chronotaxicity of cellular energy metabolism to facilitate the identification of altered metabolic states

Gemma Lancaster, Yevhen F. Suprunenko, Kirsten Jenkins and Aneta Stefanovska

Supplementary Information

Supplementary Methods. Conditions for chronotaxicity

A chronotaxic system is a nonautonomous dynamical system, with dynamics characterised by a time dependent point attractor. Considering the model of coupled oscillators,

$$\begin{aligned} \dot{\varphi}_{GO} &= \omega_{GO} + \varepsilon_1 \sin(\varphi_{GO} - \varphi_{MO}) - \varepsilon_4 \sin(\varphi_{GO} - \omega_G t) + \sigma\eta(t) \\ \dot{\varphi}_{MO} &= \omega_{MO} - \varepsilon_2 \sin(\varphi_{MO} - \varphi_{GO}) - \varepsilon_3 \sin(\varphi_{MO} - \omega_O t) + \sigma\eta(t), \end{aligned} \quad (1)$$

it is reasonable to introduce a numerical test for chronotaxicity, based on synchronization to external drivers. Synchronization was tested between all trajectories φ_j calculated from the model (1). All simulations were performed by integrating the full system of equations (1) using a Heun integration scheme with a time step of 0.1s. Trajectories were considered synchronized if $|\varphi_i - \varphi_j| < 2\pi$ for the final 5,000 seconds of a 10,000 seconds time series (to remove transients). Seven types of dynamics were found:

- A - GO synchronized with glucose, MO synchronized with oxygen, GO & MO not synchronized. **Both GO & MO are chronotaxic**
- B - GO synchronized with MO, both synchronized with glucose. **Both GO & MO are chronotaxic**
- C - No synchronization. **Neither GO nor MO are chronotaxic**
- D - GO synchronized with MO, both synchronized with oxygen. **Both GO & MO are chronotaxic**
- E - GO synchronized with MO. Both oscillators experience multiple phase slips. **Neither GO nor MO are chronotaxic**
- F - GO synchronized with glucose, MO not synchronized with oxygen, GO & MO not synchronized. **GO is chronotaxic, MO is not chronotaxic**.
- G - GO not synchronized with glucose, MO synchronized with oxygen, GO & MO not synchronized. **GO in not chronotaxic, MO is chronotaxic**.

It should be noted that, when using the inverse approach to test for chronotaxicity, false positives may be given in certain cases. In a perturbed system with a time-dependent point attractor, it is possible that the distance to a time-dependent point attractor may temporarily increase, or only decrease. Whilst the former scenario is technically not chronotaxic, these cases will not be distinguished by inverse approach methods in cases where temporal increase of deviations occurs at small time scales, and such deviations will not influence methods based on DFA.

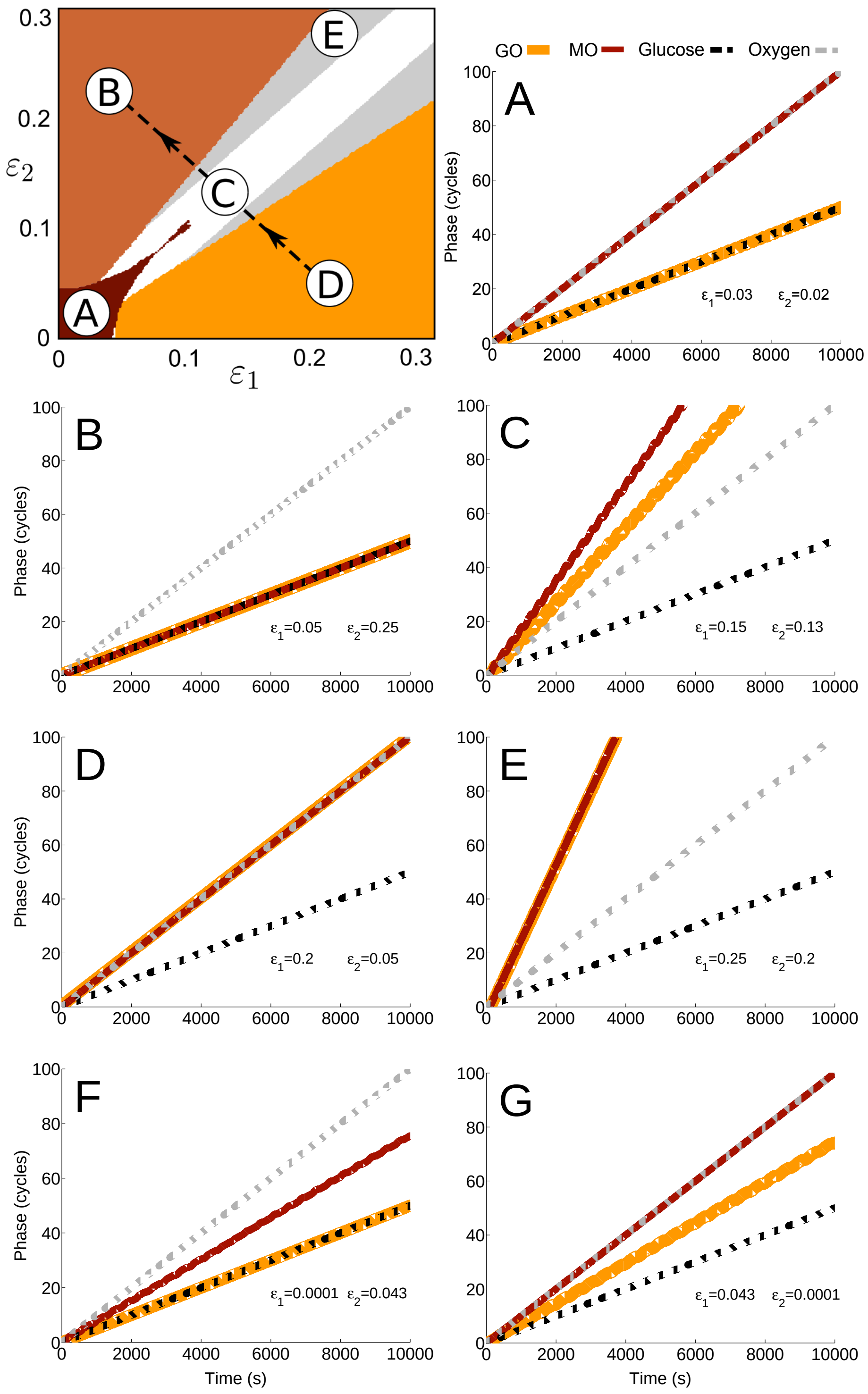

Supplementary Figure S 1: Phase trajectories of the 7 types of dynamics which have been observed in the model shown in Eq. 1. Regions F & G are not shown, see Fig. S3f. In all cases $\varepsilon_3 = \varepsilon_4 = 0.025$.

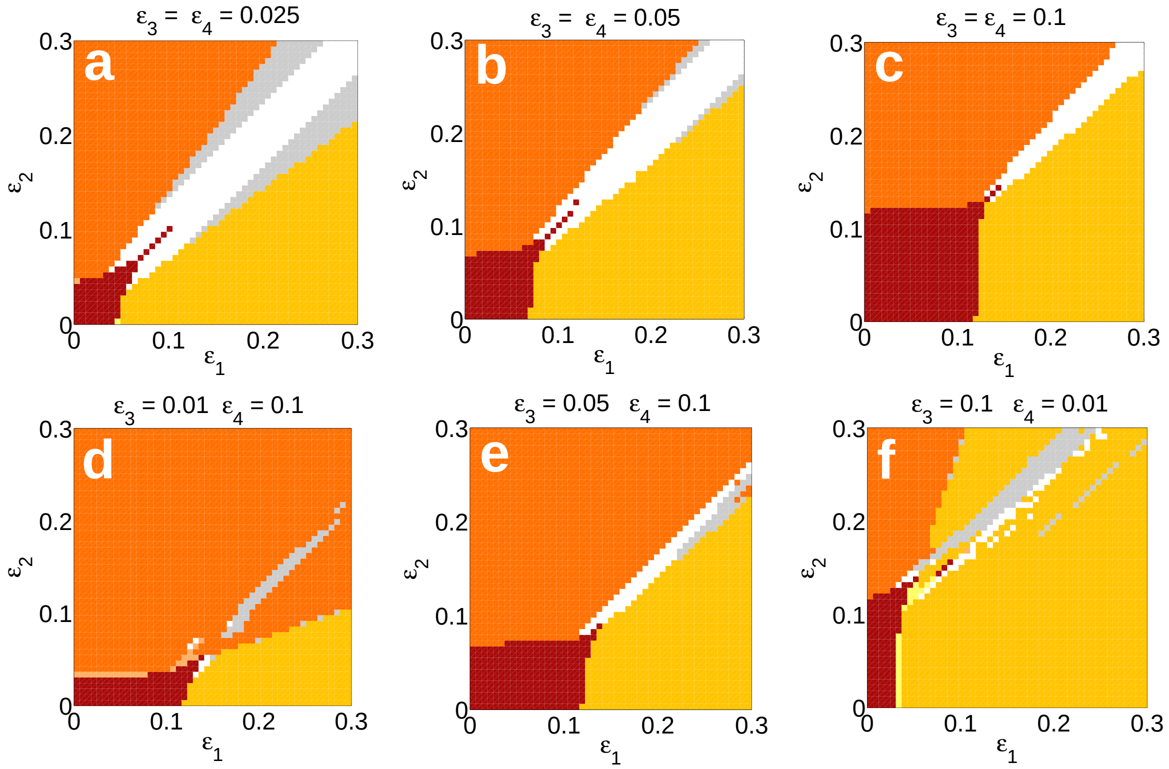

Supplementary Figure S 2: Changing modulation from glucose and oxygen, $\varepsilon_3 = \varepsilon_4$. Glucose and oxygen drivers may influence the system equally, as in Figure 4B in the main text, but their values can change. **a** $\varepsilon_3 = \varepsilon_4 = 0.025$. **b** $\varepsilon_3 = \varepsilon_4 = 0.05$. **c** $\varepsilon_3 = \varepsilon_4 = 0.1$. With increasing influence of drivers, chronotaxic areas increase. Here **d** and **e** represent a state in which the glucose driver has a larger influence on the system than the oxygen driver, i.e. $\varepsilon_4 > \varepsilon_3$. **f** represents a state in which the oxygen driver has a larger influence on the system than the glucose driver. In all cases $\omega_G = 2\pi/200$ and $\omega_O = 2\pi/100$.

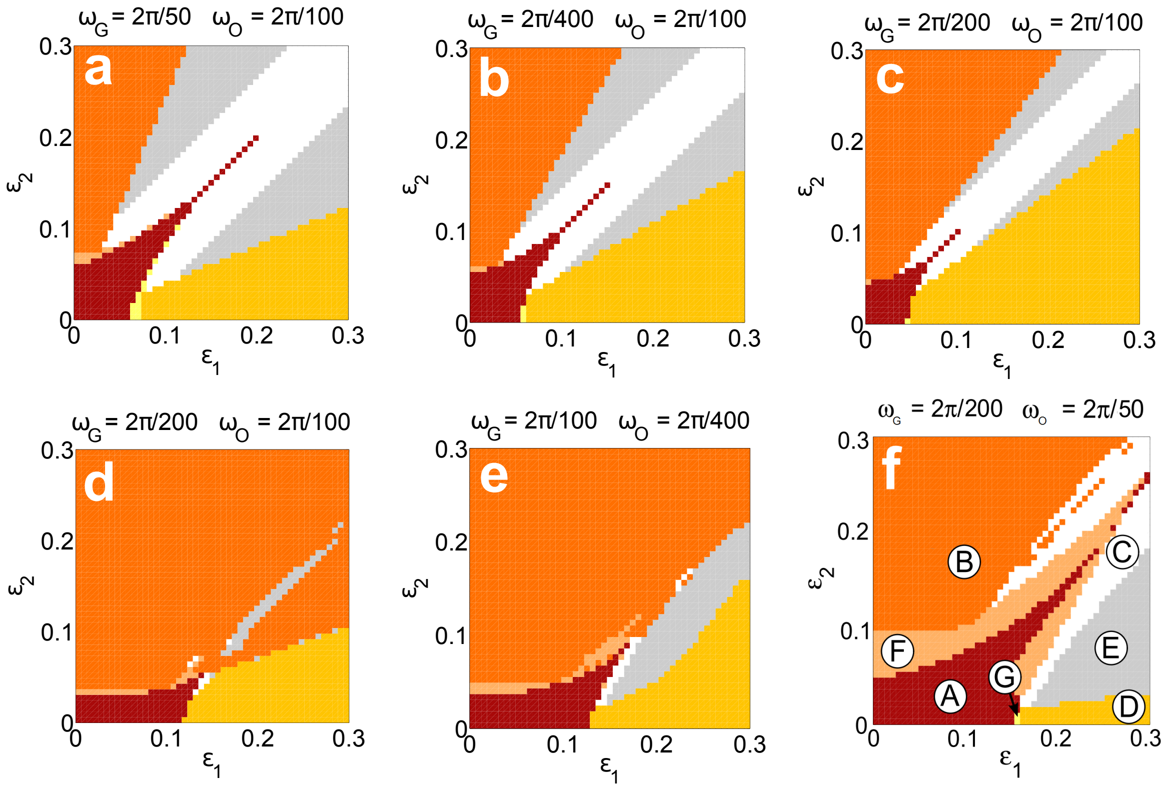

Supplementary Figure S 3: Effects of changing frequencies of GO and MO when $\varepsilon_3 = \varepsilon_4$ and $\varepsilon_3 \neq \varepsilon_4$. Different natural frequencies of oscillations may be considered, for example in different cell types. Changing frequency detuning will affect regions of chronotaxicity. In **a**-**c** $\varepsilon_3 = \varepsilon_4 = 0.025$. **c** shows the chronotaxicity plot for the system as considered in the main text, with natural frequencies $\omega_G = 2\pi/200$ and $\omega_O = 2\pi/100$. **a** shows the effects of increasing ω_G. **b** shows the effects of reducing ω_G. In **d**-**f** $\varepsilon_3 = 0.01$ and $\varepsilon_4 = 0.1$. It is possible to model a case where the frequency of GO may increase due to upregulated glycolysis, while the frequency of MO may decrease due to mitochondrial dysfunction. **d** 'Altered' natural frequencies in the case where glucose is the dominant driver. **e** Substrate dependencies as in **d** but with faster glycolytic and slower mitochondrial oscillations. **f** Effect of fixing the glycolytic frequency and increasing the mitochondrial frequency.

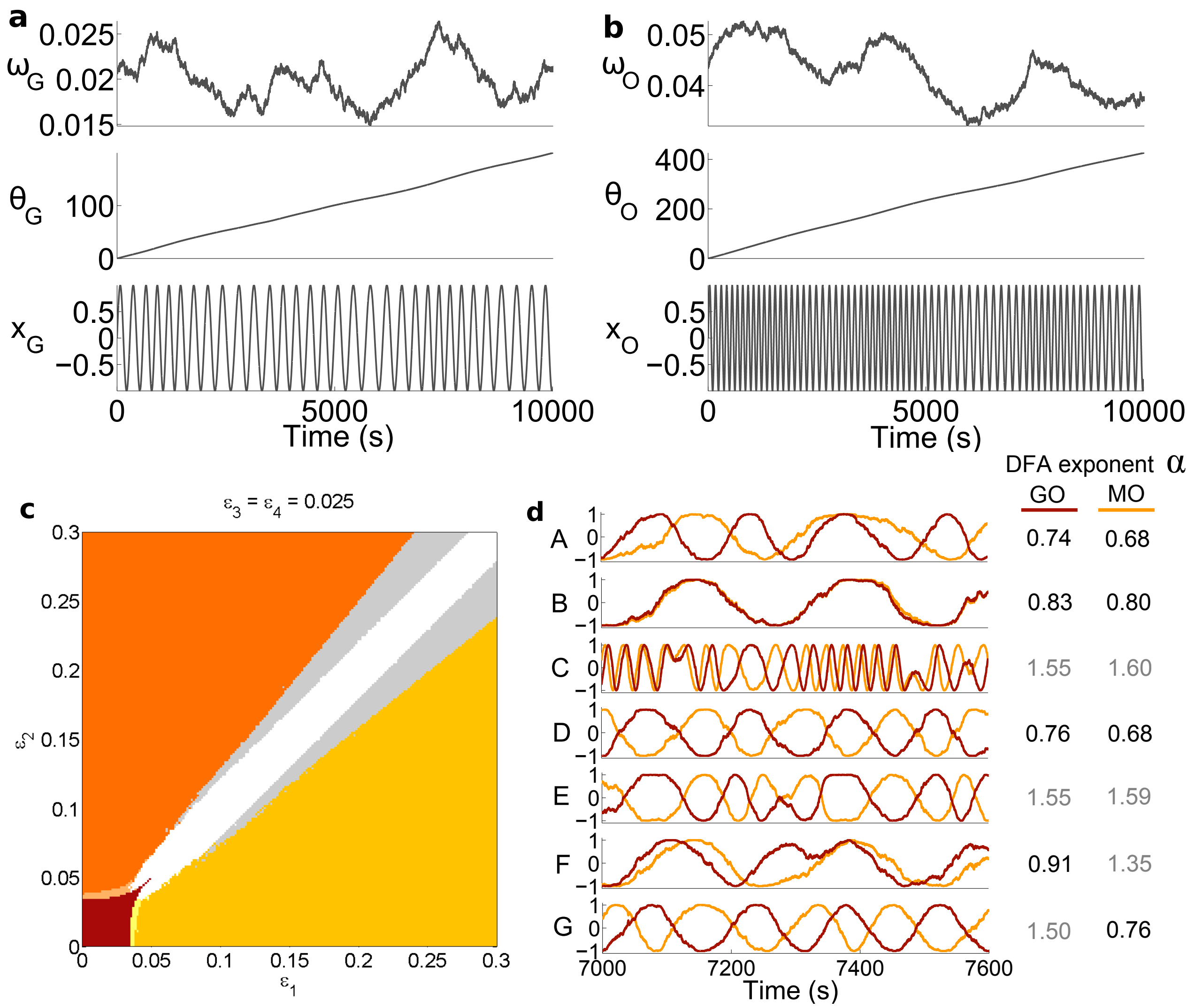

Supplementary Figure S 4: Time varying frequencies of drivers. **a-b** To demonstrate applicability of the methods of detection of chronotaxicity via the inverse approach in real data, here we allow the frequencies of the drivers to vary in time. Thus, the phases of the drivers in Eq. 1 become $\int \omega_{G/O}(t')dt'$ instead of $\omega_{G/O}t$. We model these drivers as a slow sinusoid modulated by white Gaussian noise, and allow their period of oscillation to vary in the ranges: GO = 4 - 7 minutes and MO = 2 - 3 minutes. This gives ranges for ω_G of 0.0151 - 0.0264, and ω_O of 0.0352 - 0.0522. First, the drivers were generated with a modulation frequency of 0.0003 Hz, $x_{G/O} = \sin\theta_{G/O}$ (top). **c** The chronotaxicity plot for trajectories obtained from the model is shown with driving of variable frequency (bottom). Chronotaxicity was tested as described in the main text. We note that, for the frequency ranges described, chronotaxicity can be detected in the same regions as described before, for all possible couplings of ω_G and ω_O. **d** Example 600s trajectories obtained from the model in regions A-G (defined in Fig. S1). Signals show $\sin(\varphi)$. A - In this region both GO (yellow) and MO (red) are synchronised with their drivers but not with each other, therefore the lines shown correspond to the behaviour of the external drivers. In subsequent plots, it can be seen how the signals deviate from, or synchronise with, the drivers depending on the region. DFA exponents are shown for the full time series (10000 seconds after removal of transients). The system is chronotaxic if $0.5 < \alpha < 1$ (shown by black numbers), and non-chronotaxic if $\alpha > 1$ (shown by gray numbers).

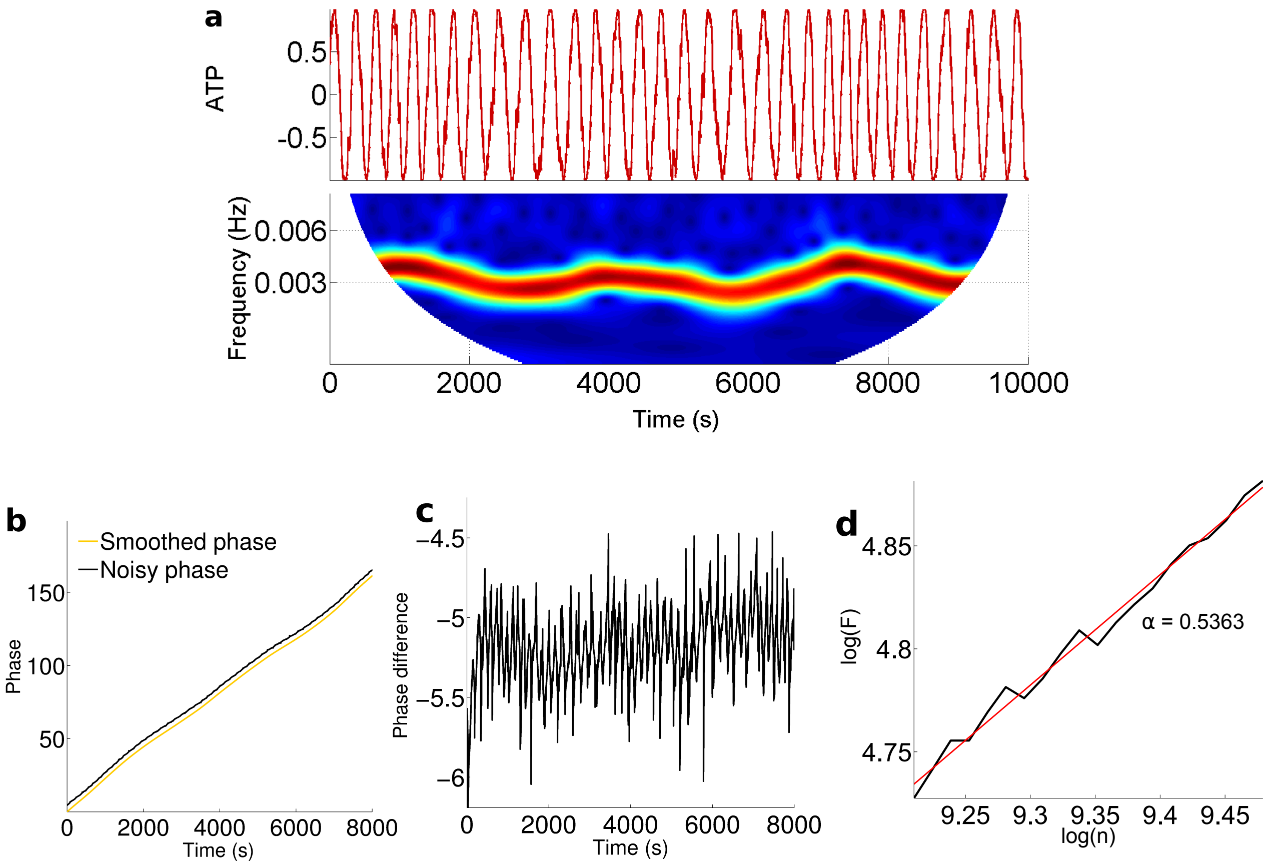

Supplementary Figure S 5: Checking chronotaxicity. **a** Example ATP signal obtained from the model (region B) and its continuous wavelet transform. **b** The phase is extracted twice, one with good frequency resolution (smoothed) and once with good time resolution (noisy). DFA analysis is performed on the **c** difference between these phases and **d** DFA exponent is obtained. In this case, $\alpha = 0.5363$, therefore the signal is chronotaxic. From the wavelet transform we can see that the frequency is around that expected during glycolysis, and thus we can say that the system is chronotaxic due to the glucose driver.
